# Biomechanics beyond the lab: Remote technology for osteoarthritis patient data—A scoping review

**DOI:** 10.3389/fresc.2022.1005000

**Published:** 2022-11-14

**Authors:** Rebecca I. Hamilton, Jenny Williams, OATech Network+ Consortium, Cathy Holt

**Affiliations:** ^1^ Musculoskeletal Biomechanics Research Facility, School of Engineering, Cardiff University, Cardiff, United Kingdom; ^2^ Osteoarthritis Technology NetworkPlus (OATech+), EPSRC UK-Wide Research Network+, United Kingdom

**Keywords:** biomechanics, inertial measurement unit, gait, osteoarthritis, remote technology

## Abstract

The objective of this project is to produce a review of available and validated technologies suitable for gathering biomechanical and functional research data in patients with osteoarthritis (OA), outside of a traditionally fixed laboratory setting. A scoping review was conducted using defined search terms across three databases (Scopus, Ovid MEDLINE, and PEDro), and additional sources of information from grey literature were added. One author carried out an initial title and abstract review, and two authors independently completed full-text screenings. Out of the total 5,164 articles screened, 75 were included based on inclusion criteria covering a range of technologies in articles published from 2015. These were subsequently categorised by technology type, parameters measured, level of remoteness, and a separate table of commercially available systems. The results concluded that from the growing number of available and emerging technologies, there is a well-established range in use and further in development. Of particular note are the wide-ranging available inertial measurement unit systems and the breadth of technology available to record basic gait spatiotemporal measures with highly beneficial and informative functional outputs. With the majority of technologies categorised as suitable for part-remote use, the number of technologies that are usable and fully remote is rare and they usually employ smartphone software to enable this. With many systems being developed for camera-based technology, such technology is likely to increase in usability and availability as computational models are being developed with increased sensitivities to recognise patterns of movement, enabling data collection in the wider environment and reducing costs and creating a better understanding of OA patient biomechanical and functional movement data.

## Introduction

### Research objective

Osteoarthritis (OA) is a highly prevalent global disease. With no cure, and no proven interventions able to stop its progression (1), it is a major cause of disability worldwide with exponentially increasing prevalence, alongside an increase in the older population (2). The consequences of OA impact significantly on the quality of life, ability to maintain sustainable work, and therefore, both individuals and wider economic strain (3), highlighting the urgent need for increased investment in large-scale scientific and clinical research (4).

For research purposes, several common minimally invasive movement-based measures are used to assess OA disease progression and outcomes following interventions (e.g., total knee arthroplasty). These are commonly extracted from human gait and include basic movement parameters with related ground reaction forces (GRFs), joint angles/moments, and range of motion (ROM) (1). The recommended performance measures to evaluate hip and knee OA (30 s chair stand, 40 m fast-paced walk, stair climb, Timed Up and Go, 6-minute walk) can be challenging for clinicians and researchers in standard fixed-assessment environments and lack real-life representation (5).

The incentive for larger-scale biomechanics research outputs within OA conditions is supported by the increasing availability of newly developing technologies (6). The OATech Network+ (7) is a collaborative UK-based research network developing technology solutions for OA, and identified novel and emerging technologies and technological advances should play a key role in directing OA research diagnosis, treatment, and monitoring (8).

Although the path towards remote data collection was laid before the Covid-19 pandemic, it has provided strong impetus for researchers to seek methods that enable OA research to be performed at a distance, increasing accessibility whilst minimising risks. In addition, there is strong evidence that the Covid-19 pandemic has increased acceptance of technology in healthcare from the perspective of both the patient and the clinician (9). Although a large body of work exists for biomechanical and wearable technology, there remains a lack of evidence that reviews and identifies its availability (commercial or experimental) and a lack of validation of such technology across the gold standard technology familiar to OA researchers. To date, no papers have been identified that focus on available biomechanical technologies developed specifically for use outside the laboratory or for remote use by OA researchers. Due to the evidence for increasing acceptance of the use of healthcare technology for patient disease progression and treatment monitoring, reduction in overall costs, and increase in data collection opportunities, the suitability for healthcare technology formally reviewed results is fitting (8). The potential healthcare economic savings, if appropriate technology is identified and utilised for patients, clinicians, and researchers, provide a compelling rationale to review and summarise technologies with suitable capabilities for the end user. Therefore, in this scoping review, the objective is to summarise the available technology that has been validated against the recognised gold standard technology to confirm its ability to deliver data that are comparable with established systems and technology found in traditional research laboratory settings.

### Technology background for remote biomechanics

The increased desire amongst biomechanical researchers to identify and adopt technology that can be used remotely is driven by a combination of factors including introducing easier testing environments (10). Studies that require quantified and objective human gait characteristics have highlighted the benefits associated with gathering real-world measurements; defined as being outside the laboratory, or “non-scripted” walking, due to the inability of clinical laboratories to reliably reflect daily-living measures (11). Significant increases in gait speed and acceleration are revealed from laboratory-based data when compared with free living data collection whilst using the same accelerometry collection methods (12–15), suggesting that laboratory-based performances can often differ from natural gait measures. This emphasises the need for better representation when collecting gait functional outputs with accurate reflections on real-life walking speed and ability. In parallel to these studies, the technological landscape has transformed in recent years, profoundly influencing healthcare, suggesting new possibilities for biomedical research (16).

Technologies that are currently in widespread use for collecting kinetic, kinematic, and spatiotemporal (SPT) measures can be classified broadly into two different approaches (17). These are based on data that can be gathered in either a controlled fixed environment or utilising wearable sensors (WS) that can be used freely. Fixed research facilities often employ established motion capture (MoCap) technology involving three-dimensional (3D) optical retroreflective marker-based systems with multiple video cameras (e.g., Qualisys, Vicon, Optitrack) and strain gauge instrumented force plates (e.g., Bertec, Kistler) measuring GRFs and/or pressure sensor force-resistive values (e.g., Tekscan, GAITRite). These can compute accurate 3D joint biomechanical measures using inverse dynamic mathematical models but are dependent on human accuracy of marker placement and laboratory calibration to reduce technical errors. These are considered the gold standard and regarded as the most accurate approach to collect human clinical biomechanical measures such as clinical gait analysis (18–20). Often used within human movement laboratories, inertial measurement units (IMUs) and mobile technologies present researchers with potential access to objective measures of gait in unconstrained environments (21). WS incorporating IMUs have been found with similar sensitivity to detect gait kinematic changes (22) and strong agreements for accuracy and consistency for gait SPTs when compared with optical motion capture (23). They are growing in popularity as a valid alternative to the more expensive and fixed systems with more environment flexibility and range of measures (24, 25).

Body-worn WS systems generally include the use of an accelerometer and have been recently reviewed as the most common technology for monitoring knee OA patients (26). IMU systems in regular use (e.g., XSens, Wearnotch), collate information gathered from three-axes accelerometers, gyroscopes, and magnetometers within each sensor. These provide raw data that are computed into kinematic outputs (joint angles, segment velocity, segment acceleration, etc.) based on the subject calibrated model and subsequently into meaningful gait SPT parameters (25). Each type of WS system has individual capabilities, and although considered to provide a lower level of reliability compared with optical motion tracking, measurements have still been accepted as clinically valid (24). The use of more portable equipment with clinically accepted accuracy levels allows a better integration of gait analysis into clinical routines (27). With IMU research growing at an exceptional rate, the growth of available parameters to analyse OA patients has, therefore, expanded based on computed individual IMU features (28)*.* The evaluation of different global commercially available IMUs suggests that selection should be dependent on the requirements of the research question, due to the potential array of parameters and collection methods, resulting in limitations within standardised IMU protocol methods (29).

There are increasingly available supplies of lightweight, portable, and accurate tools for remote measurements and monitoring due to the evolution of technology methods for data integration and hardware sensing techniques (30). Examples are stick-on skin gauges (31) and self-functioning textiles for gloves, garments, and socks that can record contact forces and physiological signals (32). Force-resistive technology is used often within laboratory environments for OA movement biomechanical analysis; however, as a remote technology, it is mainly used for physical activity and sports monitoring (33, 34).

The evolution of more powerful algorithms to convert data gathered by sensors that are clinically meaningful has also accelerated the use of mobile technologies in clinical research (35). Their level of acceptance within patient and health-related contexts has increased with their usage (32)*.* Also, the increased opportunities for researchers, and advances including wireless connectivity, real-time information, and advanced visualisation, have led to this technology penetrating the consumer market (36), with individualised measurement now made possible (37). Importantly, current evidence suggests that technology is creating a positive impact on OA treatment (38), allowing patients to manage their own condition and record their own outcome measures, whilst motivating and informing users in real time.

The development of Red Green Blue-Depth (RGB-D) sensing camera technology, in particular the launch of the Microsoft (Microsoft Corporation, United States) X-Box Kinect camera in 2010 and other commercially available products, including Asus Xtion Pro (ASUSTeK Computer Inc. United States), Intel RealSense (Intel Corporation, United States), and Orbbec (Orbbec 3D Technology International Inc., United States), is perhaps one of the most significant developments in the field of biomechanics and clinical research, offering a cost-effective markerless solution to overcome the limitations of marker-based motion capture. A review of the use of RGB-D sensors for musculoskeletal health monitoring has revealed a lack of validity assessment along with limitations (such as limited camera depth information), although models for 3D joint parameters are found to be acceptable (6). Despite these limitations (39), advances in markerless motion capture will likely change the future of data collection in biomechanics (40). These systems offer the potential to deliver an alternative approach with practical benefits for both fixed and WS due to the ability to capture data in any environment where cameras can be set up (41, 42).

Due to the growing prevalence of technological development, use, and acceptability within clinical research, the need for a formal review of the validated tools available to researchers is evident. By reviewing these recent technological developments, an indication of the future direction of remote OA research can be established with informed evidence for the appropriate tools available.

## Methods

A scoping review format was selected to synthesise research and technological developments in this field. This approach would review and summarise available evidence as a preliminary and structured assessment, whilst allowing an overview on the extensive topic. The approach was based on the Preferred Reporting Items for Systematic Reviews Scoping Review Protocol (PRISMA-Scr) (43), which was revised by the research team and members of the OATech+ Network Operations Group.

The review was conducted following a five-step process: definition of the research question, creation of a research strategy to identify relevant studies for inclusion, study selection, collation and charting of results, and analysis and reporting of results.

### Definition of the research question

To address the research objective, the primary research question of this review asked the following:

1. What technologies are available for gathering biomechanical and functional data for OA research purposes outside of a laboratory setting?

The review also asked additional secondary research questions:

2. Is the identified technology validated against an existing gold standard technology?
3. Is this technology suitable for use in a remote context, and if so, is it portable, partly remote, or fully remote (see Table 2)?
4. Which of the technologies can be identified as commercially available and therefore available for the researcher to acquire?

Papers were included only if the technology itself was the subject of the research and there was evidence of validation through human testing. This review aimed to identify available technologies used within different settings to address the above questions and provide a narrative overview.

### Search strategy

The search strategy was designed by the core research team with the support of a subject librarian and a specialist researcher. A broad literature search was undertaken in three main databases, Scopus, Ovid MEDLINE, and PEDro, using an individual search strategy for each. Grey literature searches and reference list scanning were undertaken manually. All search criteria and search dates are listed in the supplementary material.

All articles were uploaded to EndNote software (V20.1.0.15341) where duplicate titles were removed. Title and abstract screening were completed by author (JW) and full-text screening carried out by two authors independently (JW and RIH), with any conflicting views being discussed and agreed upon.

### Study selection

**Figure 1:**
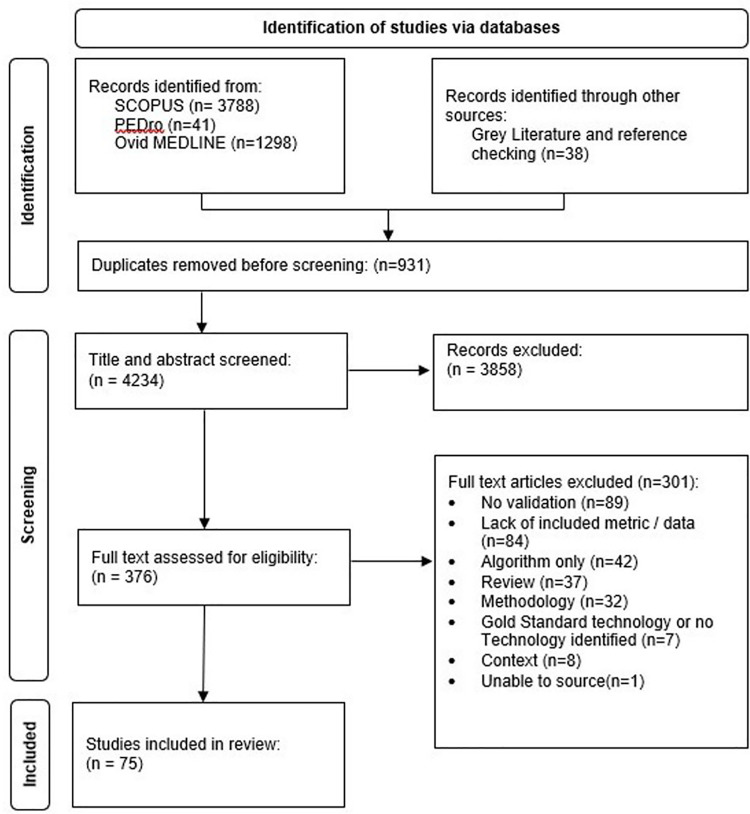
PRISMA flow diagram (2020) for new systematic reviews included searches from listed databases, registers, and other sources.

Of the 5,165 papers originally identified, 376 papers were eligible for a full-text review after duplications, and first-screen abstracts were removed (Figure 1). The papers were selected only if they met the following inclusion criteria:

(a) The focus of the study included an identified technology capable of measuring relevant biomechanical or functional parameters recognised as characteristic for OA, for example SPTs, gait, force, or pressure. Parameters that were not considered relevant for OA research, for example vertical drop jump, were excluded.
(b) The study described the results of validation of the technology against gold standard laboratory grade equipment (defined in Table 1).
(c) The study was an original article in a peer-reviewed journal and published between the period 2015 and 2021. This date range was selected due to the rapid pace of technological development.
(d) The study was written in the English language. As the OATech+ Network is an English-speaking network operated in the United Kingdom, only papers published in English were included.
(e) The study gathered data on live human participants. No conditions were excluded, and the determining factor was the parameter determined in (a).
(f) The study demonstrated the capability of the technology to collect data outside of a fixed laboratory setting. The study description of the technology’s capability of adoption remotely/portably was considered sufficient. A traditional fixed laboratory setting was considered where there was fixed equipment, for example, multi-camera systems/embedded floor force plates.

**Table 1:** Definition of gold standard for the purposes of the current study included commercially available products in widespread use.

Motion capture, video, and optical tracking equipment	Vicon (Vicon Motion Systems Ltd.), Qualisys (Qualisys AB, Sweden), Optitrack (Naturalpoint Inc., United States), Optotrack Certus (Northern Digital, Canada), NDI Polaris Vega (Northern Digital, Canada), SmartDX (BTS Bioengineering Corp., United States), Cortex (Motion Analysis Corp., United States)
Instrumented force plates, treadmills, and walkways	Zeno (Protokinetics LLC, United States), Gaitrite (CIR Systems Inc., United States), NeuroCom SMART Balance Master (NeuroCom International Inc. United States), Bertec (Bertec Corp, United States), AMTI (Advanced Mechanic Technology Inc., United States), Zebris (zebris Medical GmbH, Germany)
Previously validated human movement IMU systems including insoles	Xsens (Xsens Technologies BV, Netherlands), Medilogic (T&T Medilogic Medizintechnik GmbH, Germany), InertiaCube (Intersense Inc., United States), Wearnotch (Notch Interfaces Inc., United States), APDM Opal (APDM Wearable Technologies Inc., United States), Parotec (Paromed gmbh & Co., Germany)
Standard clinical tools	Manual goniometer, electro-goniometer, radiography
EMG systems	Delsys EMG (Delsys Inc., United States)

IMU, inertial measurement unit.

Papers were excluded if their study focus was based on algorithm models under development without an analysis of the hardware technology and its data-collecting abilities. Papers were excluded if the technological development was not available as a complete system or did not contain an available component ready for data collection use. Papers were also excluded if the technology under review was already considered gold standard and the paper was demonstrating its existing use or a new application.

### Collation of results

Data from each article were collated (Table 3), including author, title, technology in use, parameters gathered, availability, for example, commercially available, or experimental only, and the degree to which the technology was intended for remote use (Table 2). The technologies were then identified by type into four main categories. Technology was also reviewed by using recorded parameters, by the degree to which it can be used remotely, a separate table of commercially available technology as a subset of the results is also presented.

**Table 2:** Definition terms for degree of technology remoteness.

Portable	Requires a static research-suitable environment that could be a clinic or community setting where data are gathered at a defined location.	Requires specialist trained users to operate whilst data are gathered, cannot solely be participant operated
Part remote	Able to operate in most environments but has some environmental requirements, e.g., range, connectivity, wired, etc.	Requires specialist trained users to support set-up/harvest data, but data can be gathered without any specialist.
Fully remote	Able to operate in any environment or setting, e.g., home, clinic, and outdoors. Participant is unrestricted and unobserved during data gathering.	Capable of being used and managed by a non-specialist individual (or participant) with minimal support/training during data gathering.

**Table 3:** Master table of technology results divided by type, metric, application, and commercially available status.

Author and date	Tech type	Tech description	Metric	Application	Commercially available
Albert et al 2020	Cameras	Azure Kinect and Kinect V2, 3D depth-sensing camera	A	Part remote	No
Alford et al 2020	Wearables—Other	iPhone smartphone application used as a level	C	Remote	No
Amitrano et al 2020	Insoles-Platform Wearables—Other	SWEET Sock, e-textile sensor sock connected to a phone application	A B	Part remote	No
Amman et al 2020	Wearables—IMU	BioStamp, adhesive patch with accelerometer and gyroscope (IMUs)	A	Part remote	Yes
Anwary (a) et al 2018	Wearables—IMU	MetaWearCPro, IMUs with a smartphone application	A	Part remote	No
Anwary (b) et al 2021	Wearables—IMU	MetaWearCPro, IMUs with a phone application	A	Part remote	No
Aqueveque et al 2020	Wearables—IMU	IMU with a phone application	A	Part remote	No
Arne de Brabbandere et al 2020	Wearables—IMU	Samsung Galaxy J5 phone with IMU input and ML model pipeline	A	Part remote	No
Asadi et al 2020	Cameras	Kinect V2 for markerless MoCap	A	Part remote	No
Ashar et al 2017	Wearables—Other	Ultrasonic sensor network	A	Part remote	No
Bai et al 2020	Wearables—Other Wearables—IMU	Sony Move motion controller, Nintendo Wii console, Samsung Galaxy SII smartphone, and IMUs	A	Part remote	No
Barreira et al 2020	Cameras	Kinect V2 for markerless MoCap	A	Part remote	No
Bell et al 2019	Wearables—IMU	IMUs with interACTION smartphone application and web-based portal	A	Part remote	No
Bethoux et al 2018	Cameras	Kinect V2 with Echo5D custom-made software	A	Part remote	Yes
Bolanos et al 2020	Insoles—Platform	Shoe insole using force sensitive resistors and a time-of-flight camera	B	Portable	No
Bonnet et al 2015	Cameras Insole—Platform	Kinect sensor (with marker-based tracking system) and Wii balance board	B C	Part remote	No
Chen at al 2016	Insoles—Platform Wearables—Other	SmartShoe with an insole sensor	A B	Part remote	No
Cui et al 2016	Insoles-Platform Wearables—Other	Wearable Gait Lab using an underfoot force sensing unit, a joint angular and EMG sensing unit, and an Android smartphone application	A B D	Portable	No
Donath et al 2016	Wearables—IMU Wearable—Other	RehaGait, IMU set with the use of Rehawatch software	A	Part remote	No
Guess et al 2017	Cameras	Kinect V2, 3D depth-sensing camera	A	Portable	No
Haque et al 2021	Wearables—IMU Insoles—Platform	Invensense IMUs, exoskeleton with IMU system	A B	Portable	No
He et al 2019	Insoles—Platform	OpenGO (wireless shoe insole) with a Moticon smartphone application	B	Portable	Yes
Hsieh et al 2019	Wearables—Other	Smartphone with an accelerometer	A	Part remote	No
Islam et al 2020	Wearables—IMU	MoJoXlab software with generic IMUs	C	Portable	No
Jagos et al 2017	Insoles—Platform	Eshoes using a shoe insole	A	Part remote	No
Jarchi et al 2016	Wearables—IMU	e-AR, ear-worn IMU with associated algorithms	A	Part remote	No
Kanko et al 2021	Cameras	Theia3D Markerless system	A C	Portable	Yes
Kayaalp et al 2019	Wearables—IMU	Bosch Sensortec, IMUs	C	Portable	No
Khoo et al 2017	Insoles—Platform	Walk Even, shoe insole	A	Part remote	No
Kim et al 2017	Cameras	SmartGait, smartphone camera, and application	A	Part remote	No
Koiler et al 2021	Wearables—Other	Mtrigger, an adapted EMG sensor	D	Portable	No
Lanzola et al 2020	Insoles—Platform	SensFloor, carpet device	B	Part remote	Yes
Leal-Junior et al 2019	Insoles—Platform	3D-printed insole	B	Portable	No
Lee (b) et al 2020	Wearables—IMU	Skin-mounted IMU sticker	C	Part remote	No
Lefeber 2019	Wearables—IMU	Physilog IMU and GaitUp platform (software)	A	Part remote	Yes
Li et al 2020	Wearables—IMU	IMU with an Android smartphone application	C	Part remote	No
Littrell et al 2018	Insoles—Platform Camera	Wii balance board, video, and Kinovea motion tracking software	A B	Portable	No
Liu (a) et al 2017	Wearables—Other	Gazelle, IMU accelerometers, and algorithm with an online application	A	Remote	No
Liu (b) et al 2018	Wearables—IMU	Inertia Link, IMU	A	Part remote	No
Liu (c) et al 2019	Wearables—Other	Mini accelerometers	A	Part remote	No
Lubetzky et al 2019	Wearables—Other	Oculus Rift HTC Vive, virtual head-mounted display	A	Part remote	No
Manor et al 2018	Wearables—Other	Smartphone application	A	Remote	No
Moon et al 2017	Wearables—IMU	BioStamp, IMU skin-mounted plaster	A	Part remote	Yes
Moore et al 2017	Wearables—Other	AX3 Axivity, wearable accelerometer	A	Portable	No
Moreno et al 2017	Cameras	PrimeSense RGB-D camera	A	Portable	Yes
Niechwiej-Szwedo et al 2018	Cameras	Leap Motion Controller, optimal tracking device	A	Part remote	Yes
Ohberg et al 2019	Wearables—IMU	MoLab POSE, IMUs	A	Portable	No
Ong et al 2018	Wearables—IMU	Invensense IMU, experimental hardware and software	A	Portable	No
Ostaszewski et al 2020	Insoles—Platform	Shoe insole	B	Part remote	No
Otte et al 2016	Cameras	Kinect V2, 3D depth-sensing camera	A	Part remote	No
Oubre et al 2020	Wearables—IMU	Shimmer IMU with a retractable string sensor experimental device	C	Portable	No
Parks et al 2019	Cameras	MO2CA iPhone 7, smartphone application	A	Portable	No
Renner et al 2021	Insoles—Platform	Loadsol placed within a commercially available running shoe	B	Part remote	Yes
Silsuspadol (a) et al 2017	Wearables—Other	Vivo X5 plus smartphone with application, worn on the hip	A	Remote	No
Silsuspadol (b) et al 2020	Wearables—Other	Samsung or Asus Android smartphone with application, worn on the hip	A	Remote	No
Smith et al 2018	Wearables—IMU	IMUs	A	Remote	No
Solanki et al 2018	Insoles—Platform	ShoeFSR, smart shoe	B	Part remote	No
Tchelet et al 2019	Wearables—Other	Encephalog, smartphone used as a wearable	A	Remote	Yes
Van Helvoort et al 2021	Wearables—IMU's	Gaitsmart IMUs	A	Part remote	Yes
Vilas-Boas (a) et al 2019	Cameras	Kinect V2, 3D depth-sensing camera	A	Part remote	No
Vilas-Boas (b) et al 2019	Cameras	Kinect V1, Kinect V2, depth cameras	A	Part remote	No
Werner et al 2020	Wearables—IMUs	Dynaport Movetest IMUs	A	Portable	Yes
Wu et al 2021	Wearables—IMU	Nushu, IMUs	A	Part remote	No
Xia et al 2017	Wearables—Other	SmartShoe	C	Part remote	No
Xu (a) et al 2015	Cameras	Kinect 2, 3D depth-sensing camera	A	Part remote	No
Xu (b) et al 2017	Cameras	Kinect 2, depth-sensing camera	C	Part remote	No
Yagi et al 2020	Cameras	3D depth-sensing camera with a OpenPose model	A	Portable	No
Yang et al 2019	Insoles—Platform	SITUG, shoe insole with a smartphone application	A B	Part remote	No
Ye et al 2016	Cameras	Kinect V2, 3D depth-sensing camera	A	Part remote	No
Yeh et al 2016	Wearables—Other	Wii remote sensor, handheld motion sensor	A	Part remote	No
Yeung et al 2021	Cameras	Azure Kinect, Kinect V2 and Orbbec Astra, and 3D depth-sensing cameras	C	Part remote	Yes
Zhang (b) et al 2017	Insoles—Platform	Sportsole, shoe insole	A B	Part remote	No
Zhang (c) et al 2021	Wearables—Other	Sensor using two smartphone applications and data acquisition module (motion sensor, microcontroller unit, power supply, and Bluetooth)	C	Part remote	No
Zhu et al 2021	Cameras	Azure Kinect, 3D depth-sensing camera	C	Part remote	Yes
Zugner et al 2019	Wearables—IMU	Gaitsmart, IMUs	A	Part remote	Yes

A, kinematics and SPTs; B, kinetics and SPTs; C, joint angles/ROM; D, electromyography; IMU, inertial measurement unit.

## Results

### Study selection

Figure 1 illustrates the literature search and exclusion criteria with further exclusion details in supplementary material (Table 2). Following the search strategy within three databases (Methods Section “Search strategy”), 376 full articles were assessed for eligibility, from which 75 were identified for inclusion within the final screen (Figure 1).

### Technology themes

The remaining articles were assessed and recorded categorically depending on the broad technology type, location of use, metrics measured, and their commercial availability (Table 3).

**Figure 2:**
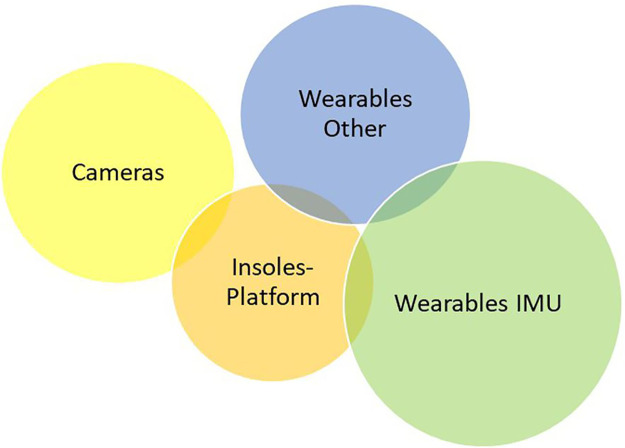
Technology results described by type category within a Venn diagram demonstrating several technologies measuring more than one category type (10.7%). IMU wearables were used the most in 25 technologies (30.1%), wearables of other varieties were used in 20 technologies (24.1%), cameras were used in 21 technologies (25.4%), and insoles or platforms were used in 17 technologies (20.5%).

**Figure 3:**
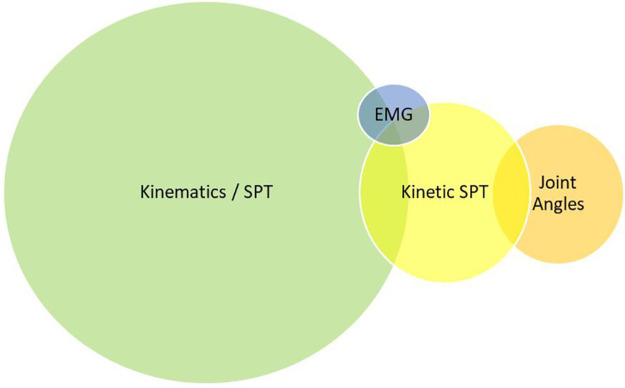
Technology results described by metrics measured within a Venn diagram demonstrating several technologies measuring more than one metric category (12%). Kinematics and SPT measures were used the most in 54 technologies (64.3%), kinetics and SPT measures were used in 16 technologies (19%), kinematics measuring joint angles and ROM were used in 13 technologies (15.5%), and electromyography measures were used in 2 technologies (2.4%).

**Figure 4:**
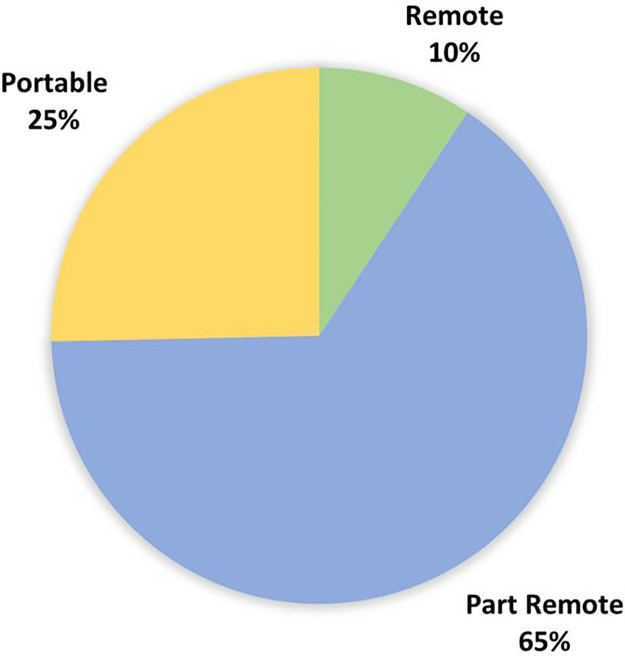
Technology results divided by application categories based on the criteria described in Table 2. Most technologies were categorised as part remote (65%), whilst 19 technologies were categorised as portable (25%), and 7 technologies were identified as fully remote (9%).

Based on the range of results, the technologies were categorised into four main technology types, with some meeting classification criteria for more than one category. A large percentage of technologies were identified as wearable devices and were divided into those consisting of “IMUs” and “other wearables.” The remaining two categories were “cameras” and “insoles/platforms” using force-resisting sensor technology. Figures 2–4 demonstrate the division as well as the overlap of the technology type, metrics recorded, and location. “IMU wearables” (Figure 2) are the most prominent technology type, “kinematics with SPTs” (Figure 3) is the most prominent metric recorded, and “part-remote” (Figure 4) is the most used application of the technology screened.

The description of the metrics used was also divided into four main categories described below (Table 3), with the metrics being divided based on kinetic and kinematics as well as further divided into use of joint angle/ROM, SPT, and Electromyography (EMG) data collection. The application of the technology was divided into three main categories based on the definitions described in the Methods Section “Collation of results” (Table 2), and the commercially available status of the technology was based on the information available in the article, with further details on these provided in Table 4.

**Table 4:** Identified commercially available technology with related corresponding information and study reference in which it was evaluated.

	Description	Metric	Location	Study Reference
BioStamp https://www.mc10inc.com/	Skin adherent sensor patch with an accelerometer and a gyroscope (IMUs)	Tri-axial linear/angular motion, ROM, joint angles, and gait SPTs	Part remote	(44, 45)
Echo5d from Atlas5D https://atlas5d.com/our-technology/	Ambient measurement system—non-wearable activity monitoring	Gait SPTs—ADLs	Part remote	(46)
Encephalog from Mon4t https://mon4t.com/movement/	Smartphone app (integrated tri-axial accelerometers and gyroscopes)	Gait SPTs—specifically in relation to TUG parameters	Remote	(47)
GaitSmart https://www.gaitsmart.com/	IMUs with proprietary software	Gait SPTs, ROM, kinematic parameters	Part remote	(48, 49)
Loadsol https://www.novelusa.com/loadsol	In shoe worn insole device	Plantar peak force	Part remote	(50)
McRoberts Dynaport MoveTest https://www.mcroberts.nl/products/movetest/	Single IMU belt worn device	Gait SPTs	Portable	(25)
OpenGo by Moticon https://moticon.com/opengo	OpenGO (wireless shoe insole) with a Moticon smartphone application	Kinetic parameters (KAM), gait SPTs	Portable	(51)
Physilog GaitUP https://research.gaitup.com/physilog/	Two Physilog IMUs and proprietary Gaitup software system	Gait SPTs	Part remote	(52)
SensFloor https://future-shape.com/en/gait-recording/	Capacitive sensor embedded flooring with recording outputs	Gait SPTs	Part remote	(53)
Theia3D Markerless https://www.theiamarkerless.ca/ In conjunction with Qualisys (Qualisys AB, Sweden) Miqus cameras	Markerless motion capture software for processing of camera-generated video to produce 3D kinematic data (segments and rotation matrices) that are ready for analysis	Kinematic SPTs, segments, and angles	Portable	(54)

IMU, inertial measurement unit; ROM, range of motion; SPT, spatiotemporal.

Camera and pose estimation developments were demonstrated in a vast array of the results and particularly, 14 studies used either Azure Kinect or Kinect V2. As Kinect V2 is now retired, the Azure Kinect version is likely to be found in future studies for measuring SPTs and joint angle calculations based on these validation studies. With two studies comparing Azure Kinect with the Kinect V2 predecessor (55, 56), the Azure Kinect is described as revealing promising results for full-body spatial parameters with accuracy, although with a note of caution on depth camera angles. Given the strong Azure Kinect and V2 comparison results (Pearson correlation coefficient, *r* = 0.8–0.98) and its usability in home environments, it is expected to be in future studies for patient and at-home exercise monitoring for several at risk groups. As field-of-view limitations and depth camera complexities were discussed in more than 10 of the camera-based studies, many studies still revealed good to excellent correlations and accuracy values for SPT and joint angle-based parameters, but also advised caution (57, 58).

IMU was the single most prominent technology type found within the studies (25 technologies, Figure 2). The growth in this technology is phenomenal with miniaturised devices that can be embedded into other hardware and attached to the body for data collection. This is evident from the examples of several IMU technology types such as skin adherents (44), body attachments on the hip and wrist (59–61), embedding with smartphone-based applications (62), as well as waterproof technology (63). Alongside good reliability and accuracy results (60, 64, 65), its main advantage lies in the ease of data collection, as demonstrated by the number of people using it, and as testified by the results of the studies.

Other wearables were demonstrated in good numbers in the results when embedded with smartphone applications already in use, and therefore, capitalising on an already established platform, both for the researcher/clinician and for the user. These, therefore, have good usability rates within the study results (66, 67) based on the knowledge already acquired and demonstrated as six of the determined fully remote technologies identified within this technology type category. There are also some identified needs for making improvements in specific movements such as faster walking (68) or for specific patient population groups (69).

### Commercially available technology

Within the 75 articles, 57 different technologies were identified, and a majority of these were experimental or made use of a commercially available component, for example, IMU, smartphone, activity monitor, RGB cameras, virtual reality (VR) headsets, optical tracking devices, or video game hardware components. Whilst some of these components are commercially available, only technologies that are commercially available as a complete system (both gathering and displaying results for their described use) appear in the results in Table 4. A total of 12 papers referred to the use of the Microsoft Kinect V2 camera, now retired and therefore does not feature in Table 4. One paper referred to a previously commercially available product Hasomed RehaGait (HASOMED GmbH, Germany), retired as of December 2021, and therefore excluded from Table 4. The current or future availability of each of these technologies has not been verified.

## Discussion

With a considerable number of results and experimental technology under development, there is growing interest and feasibility for research in this field from numerous groups. Almost 75% of the technology have been identified as participant wearable technology—body fixed or shoe worn, giving rise to both individual needs of data collection methods and types and their range of potential in different uses. The remaining 25% focused on camera technology with the growing prevalence of markerless MoCap (54) in use. Although this is predominantly still within laboratory settings, it comes with a degree of portability. A total of 75% of the technology identified is focused on the lower limb due to the prevalence of gait and SPT measures used within the data collection and reflects the majority of OA research focus on the lower limb. Gait SPTs are the most common and is, therefore, expected to be the most valuable; less valuable were the two studies using EMG technology.

### Technology types

#### Wearables—IMU

The range of methods, protocols, population groups, and overarching contexts used within the IMU studies indicate the extent of their potential as well as supporting previous observations stating their considerable commercial availability. Most studies using IMUs were interested in SPT outcome measures, in agreement with previous OA research (70), as a highly useful clinical evaluation tool. Mainstream use of IMU technology was found to be prevalent for studies collecting kinematic parameters in clinical research and rehabilitation settings (71), with a small number of IMU studies focussed on predicting joint angles and ROM measures (72), which were mainly developmental in nature. Oubre et al. was the exception, using a Shimmer IMU alongside a bendable and stretchable string sensor to measure the change in string length between two anchor points (72), although not commercially available together as a system. This indicates a field of research that is cumulatively gaining interest. However, it involves increased complexities of computational predictive modelling to produce joint angle data due to the information required from three-axes data and related biomechanical models when compared with optical motion camera-based measures.

Most IMUs were demonstrated as belt or strap-worn devices. However, what emerged in two studies was a commercially available skin adherent IMU, namely Biostamp (BiostampRC, M10 Inc. United States), found to be well suited to a variety of uses, including potential granular monitoring of gait both inside and outside the clinic (45). A variety of experimental systems were found reporting on IMU data collection alongside a mobile application (62, 73) and visual user feedback (74) with good accuracy and platform outputs for joint angle measurements, demonstrating strong potential for reliable home-based rehabilitation data collection. The usability of smaller devices growing within the IMU commercial sector enables small and adaptable sensors that can be easily attached in comparison with sensors with larger hardware and more uncomfortable for the user. This is particularly observed with the BioStamp IMU used in Ammann et al. (44) and Moon et al, which is a stretchable and waterproof skin adherent and is expected to be very cost-efficient at under $10 per sensor. With the promise of reliable and accurate results for gait monitoring, this IMU, along with other skin and textile-based sensors, is likely to be highly used due to the feasibility of comfortable data collection, visual user feedback (74) with good accuracy, and platform outputs for joint angle measurements, demonstrating strong potential for reliable home-based rehabilitation data collection. The usability of smaller devices growing within the IMU commercial sector enables small and adaptable sensors that can be easily attached in comparison with sensors with larger hardware and visual user feedback (74) and platform outputs for joint angle measurements, demonstrating strong potential for reliable home-based rehabilitation data collection.

Several studies revealed positive patient acceptance and usability for IMU wearables (74–76). However, all IMUs identified in this review were considered suitable for part-remote use only, requiring expert support due to specific requirements around their placement or connectivity. Therefore, despite the evidence that they provide solutions for remote data collection protocols, the level of support required for their set-up and data acquisition means that they are unlikely to be suitable or utilised for long term at-home data collection.

The variability of IMU data collection methods and a lack of consensus with regard to the standardised measures to be adopted and IMU positioning offer researchers flexibility for application; however, it decreases the ability to compare and utilise shared data and results (62). Although recommendations have been made for standardising IMU data collection methods for SPT parameters (62, 77), further pragmatic guidelines using validated methods are required to aid future remote gait assessment where environmental unknowns will complicate data interpretation.

#### Wearables—other

This technology group is dominated by small consumer-grade devices, for example activity monitors, VR headsets or consumer smartphones containing accelerometers, gyroscopes, and cameras. The value of these technologies lies in their level of remoteness, because many of them comprise smartphone technology, and already there are common consumer devices with simple user interfaces. Six out of the seven fully remote technologies are in this category and use smartphone application software. Therefore, this is likely to be a successful route for determining the true remoteness of the data collection methods. Although growing in popularity, the current ageing population is prevalent within the OA population and affects the levels of usability and feasibility of the technology (60). This is supported by mixed results for SPT measures in the studies of this category. Although good validity was found for SPT outputs from a smartphone application with camera tools (78), many unreliable results were found for smartphone 3-axes accelerometers (68) and IMU SPT data acquisition (59). This suggests that camera-integrated systems utilise better developed technology for end result reliability. Although consumer-grade technology is widely available, only EncephaLog was commercially available as a complete solution for researchers gathering the metrics of interest (47). This highlights the fact that many of these fully remote and smartphone-based tools must see more development.

Interestingly, studies that used more than one system at a time (79, 80) revealed a focus for technology fusion applied to future research data collection as the technology improves. Both studies demonstrated the value of using smartphone application software for data filtering, processing, and outputs, providing a successful, user-friendly tool for reduced use of laboratory-based equipment. Cui et al. used portable EMG in conjunction with wearable technology to collect kinematic and kinetic data parameters. The main data used for functional parameters, however, were force sensing and IMU units. A lack of EMG sensor data collection in the study results also implies that this parameter is generally considered alongside other biomechanical parameters and less valuable information.

#### Insoles platform

Most technologies in this category measured kinetic and SPT outcomes and took the form of an insole or device placed within a standard or customised shoe in common with widely available commercial products familiar to researchers. Generally, *via* force or pressure resistive sensors, when force is applied through the plantar surface of the foot (e.g., during the stance phase of a gait cycle), a change in resistance allows the phases of gait and pressure distribution on the plantar surface of the foot within a shoe/sock/insole to be calculated. These data are then used to determine SPT outputs and can be used in conjunction with kinematic-based sensors and outputs (65, 79, 82, 83) or a camera-based technology (83, 84). These kinetic data, along with kinematic joint angle data, could be paired with smartphone software, similar to other wearable/remote technologies reported (85).

Of specific note is an experimental textile sock for analysis of gait and posture (82) that could overcome issues associated with insoles because they create an additional layer that can change the distribution of foot plantar loading (86). This would be more representative of laboratory-based activities that are usually undertaken barefoot. Also of note is SensFloor (SensFloor Gait, Future Shape GmbH, Germany) (53), a carpet product capable of recording basic SPT measures through the identification of gait phases *via* the floor sensors, which has shown good validity when compared with reference values. The carpet was identified as cost-efficient and with good potential for patient rehabilitation monitoring, although limited to a defined environment.

The commercially available technologies in this category reported were Loadsol (50) and OpenGo (Moticon Rego AG, Germany) (alongside a smartphone application) (51). Both are demonstrated as popular insole devices for gait data collection within the general market, showing good usability features. Loadsol (Novel Electronics Inc., United States) insoles demonstrated high correlation values for vertical GRFs when compared with a gold standard instrumented treadmill. When used to detect gait impulse and loading rate, they could successfully identify various comparators such as age groups and degree of walking incline, thus providing an approachable technology for monitoring force and load information for patients’ gait. OpenGo demonstrated effective data acquisition and possible use as a rehabilitative tool with auditory cues and knee adduction moment calculations, a well-known measure for OA disease progression (51, 87). Auditory feedback was administered *via* the smartphone application and demonstrated promising use for both rehabilitation training and patient monitoring within a home-based environment. It also showed great potential to integrate the technology with other wearable/remote tools for rehabilitation and data collection, strengthening the argument for developing fusion techniques with or without smartphone application.

#### Cameras

RGB-Depth cameras were found in a quarter of the results, many using the Microsoft Kinect skeletal tracker camera solution launched in 2010, with an upgraded version 2 launched in 2014. These cameras have the advantage of operating as a single-camera system where multi-camera systems are not feasible (55), for example, in clinics, field test conditions, and fitness centres, and they do not require body fixed components. Whilst widely used in research, Kinect has had mixed results in comparison with gold standard systems, showing limitations for determining SPT parameters and in terms of 3D kinematic accuracy (88–91). However, it showed high accuracy for simple kinematic measures such as 3D ROM and movement velocity (92). If combined with other systems, accuracy may be improved (84).

Markerless motion capture software is seeing growth in both research and industry settings, and “Theia 3D Marker-less” was found in this review as a prominent commercially available system. By using the optical motion camera set-up, and thus limiting the remoteness of its use, the deep learning algorithm-based system removes the need for marker-based set-ups and increases the portability of its use. Although further testing is stated as required to enable better sensitivities to environmental factors and subject characteristics, the promising and comparably accurate results of marker-based motion capture demonstrate its potential for improving the feasibility and sample size of OA patient data collection. Although it is one of the most promising developments within portable camera-based motion capture technology, the cost of the system set-up within or outside laboratory environments is likely to be more than $20,000, and a knowledge of and training in the system set-up is required, regardless of whether the motion caption camera systems are already in place. Therefore, a high degree of expertise to handle the set-up and potentially good equipment are still required. However, the cost is still less than that of standard 3D marker-based motion capture technology, and it has great potential for patient-based data collection without the need for marker placement.

Other commercially available camera and optical tracker components were found to give reliable results only for functional test outputs (Multi-Directional Reach Test, Timed Up and Go) (93) and had limitations based on errors when compared with optical tracking systems (94). Only one technology incorporating a depth camera was found to be commercially available. The Echo5D is described by the manufacturer Atlas5D (Lincoln, MA, United States) as an ambient measurement system comprising a single depth camera and bespoke software for use at home. Although it was suitable for use in a defined environment, validated use was for a single parameter—walking speed—specifically in an multiple sclerosis (MS) population (46); therefore, the use for an OA or for other musculoskeletal (MSK) clinical populations may be limited. Although all individual depth camera devices found in the results are commercially available, none, other than the Echo5D, were identified as being available as a standalone system specifically for human movement measures. However, they offer adaptive potential for research and data extraction purposes, offering significant and growing potential for the OA researcher.

Both the Kinect and the Nintendo Wii systems were developed primarily as gaming technologies for the entertainment market and were subsequently recognised by researchers for their potential. The original Kinect system (V1 and V2) has now been retired, and therefore, products incorporating it are not commercially available. Kinect V1 and V2 were superseded by the launch of the next-generation AI Microsoft Azure Kinect sensor released in 2020. Azure Kinect has a suite of applications including the Body Tracking SDK pose estimation model of human movement focused on non-gaming industries including healthcare, MSK diagnosis, and exercise evaluation (6). The Azure Kinect has been reported as demonstrating improved results for spatial measures compared with the original Kinect. Good comparison validity measures were found for finger and thumb joint angles when compared with optical systems (55, 95) as well as full-body tracking for joint angles during treadmill walking (56). However, caution is required with camera viewing angles when using a range of depth sensors for kinematic gait measurements. Considering the limitations, depth cameras are useful as a portable motion capture tool but may still require a small defined environment.

### Location/application of technology use

Freedom to use the technologies in any environment/location and their ability to be applied for a variety of uses without specialist knowledge or support, are fundamental to classifying the technologies as suitable for fully remote use. Many of the technologies in the review results lacked a method or reference for real-life, real-time assessment of remote or non-laboratory use. Therefore, most were only described as hypothetically suitable for remote use, and in some cases, no method for remote use was suggested. Equally, many studies lacked detail on how data would be recovered and analysed, for example, in real time or *via* additional processing. Additional factors such as battery life, range of use, method of data recovery, and analysis would also impact usability and availability of the data.

Very few (9%) technologies could be determined as fully remote, with two-thirds (68%) classified as part remote and the remainder (23%) portable only (Figure 4). “Portable” technology offers OA researchers additional tools to use in community, clinic, or other settings outside of the traditional laboratory and may still offer new and more cost-effective ways of gathering kinetic and kinematic metrics than those currently available. Therefore, we can conclude that the use of technology outside of the laboratory for OA research is both feasible and possible.

Most technologies that are commercially available (Table 4) were identified as “part remote” and measuring SPT parameters. This highlights the fact that trained users (patients/researchers) have an increasing number of opportunities to collect real-world data in a variety of settings and these opportunities are likely to continue growing and developing. Although small, the identification of fully remote technologies could offer researchers the potential to gain new insights into the lives of those with OA through the ability to collect data in an unrestricted and unobserved way. This increases the potential for collecting data over a longer period, enabling patterns within data to be analysed, as opposed to one-off laboratory visits.

### Experimental technologies

The results demonstrated a wide range of technologies under experimental investigation for the gathering of useful OA research data. Whilst some of these (commercially available products) were similar to the IMU or insole platforms, others suggested alternative remote approaches, for example, a reliable self-measurement hand ROM tool using the Apple iPhone (96) as well as a proposed ultrasonic sensor network system for convenient at-home gait assessment (97). These systems are complemented by findings in other work advocating the use of non-contact, low-impact sensing such as smartphone applications for the measurement of ankle ROM (98) and pulsed Doppler radar (impulse radio ultrawide band) to understand human walking patterns (99).

It is likely that further rapid development of smart wearable technologies, AI, and other technologies will gain greater focus for gait research, resulting in a paradigm shift to acquire complex data employing predictive analytics (100). It is also highly likely that further advances in gaming technology (such as VR) will be better deployed for biomedical use (101), leading to further advances in markerless data capture.

### Limitations of the study

A narrative overview of identified technologies was the primary objective of the research; however, it would be beneficial to conduct an in-depth comparative analysis within the measured technology type/parameters. Other technologies that did not meet the inclusion criteria, due to their size or operating requirements, may still be suitable for remote or community use. Most studies did not include an OA population, an aging population, or a population mixed across the socio-economic divide. Translation to an OA population may be essential for evaluation depending on the research requirements. Most studies did not evaluate intra-operator reliability, which contributes to the feasibility of translation of remote technology for use with OA patients. This also affects technology usability, a critical element for successful use of remote technology in research (102).

Quality scoring of technology could have considered the advantages and disadvantages based on economic factors, research skills and usability, environmental feasibility technical specifications, or cost (and thus practical elements that may impact usability such as weight, size, battery life, operation range, and user interface complexity). Equally, no consideration was given to the nature of the data recorded and how such data could be accessed or harvested from the device, or to the ease of analysis or interpretation of such data.

### Further research

Given the range of technology scoped, OA researchers would benefit from studying the available evidence of technology for the specific parameters and environment necessary or from conducting a pilot feasibility study. This could be incorporated into a larger-quality scoring assessment and include inter- and intra-operator reliability scoring. Increasing developments in portable-based technology will give rise to new opportunities for *in situ* OA patient data collection and broaden the field for new approaches. Because most results were reported to be of hypothetical use, outside the laboratory, or with other patient groups, there is still a need for ensuring real-life data collection accuracy and feasibility of these technologies in OA patients. The validity of the technology for the proposed purpose and the impact on both researchers and participants can then be better understood, managed, and mitigated.

## Conclusion

A wide range of potential technologies are available for the OA researcher to use outside of a traditional laboratory-based environment, including various technologies that are commercially available. Currently, they are mostly limited to the provision of gait SPT measures collected within a part-remote scenario. With IMUs as the most prominent technology used, standardised data collection methods will improve their usability for OA patient groups. The emergence of fully remote devices is likely to capitalise on the use of smartphone application technology and data fusion techniques to advance this development. Evidence suggests that new emerging technologies under development will increase the choice and availability of technology solutions for OA researchers in the future. Markerless motion capture is gaining traction in both research and industry settings (e.g., gaming technology) and vision-based approaches, with growing computational sensitivities likely to expand the feasibility of OA patient data collection. Embracing the emergence of innovative technologies offers the potential to simplify methods, influence the targeted patient group and outputs, reduce the cost of and skills necessary for data collection, and widen the locations and environments in which data can be collected. Technology that can operate remotely will facilitate the gathering of objective data and a better understanding of real-world OA and its impact on the patient.

This research has identified several technologies that can support the OA researcher currently and can provide data of differing types and quality, with IMUs identified as the most prevalent technology type in use and most likely used for the collection of SPT measures. Technology selection is only a consideration for the OA researcher, and further research to understand the impact on both researchers and participants and the feasibility of operating research projects with remote technology is required.
