# Supplementary material for: Biomechanics beyond the lab: Remote technology for osteoarthritis patient data—A scoping review
Source: Front Rehabil Sci. 2022 Nov 14;3:1005000. doi: 10.3389/fresc.2022.1005000 (PMC9701737; doi:10.3389/fresc.2022.1005000)
Supplement: Supplementary file 1 [file Datasheet1.docx]

Supplementary Material

# Search Strategy

The terms and preliminary keywords used were:

(Tech* or device or sens* or internet or web or mobile or app* or wearable) AND

(monitor* or measur* or assess* or record* or track* or captur* or evaluat* or sens*)

AND (biomechanic* or gait or kinematic*) AND

(home or communit* or remote or portable or free living)

**Table A – Tabulated Description of Search Terms**

|  | **Concept** | **Population** | **Outcome** | **Context** |
| --- | --- | --- | --- | --- |
| Tech* | Monitor* | Human | Biomechanic* | Home (may have home-based) |
| Device | Measur* |  | Gait | Community |
| Sens* | Assess* |  | Kinematic* | Remote |
| Internet / web | Record* |  |  | Free living or free-living |
| Mobile | Track* |  |  | Portable |
| App* | Captur* |  |  |  |
| Wearable | Evaluat* |  |  |  |
|  | Sens* |  |  |  |

# Completed Searches

**Ovid Medline** Run 29.07.2021 = 2084

Ovid MEDLINE(R) ALL <1946 to July 28, 2021>

1 tech*.mp. 3576986

2 exp "Equipment and Supplies"/ 1536270

3 sens*.mp. 2505843

4 "Internet of Things"/ or Internet/ or Internet-Based Intervention/ 77285

5 web.mp. 136728

6 mobile.mp. 115584

7 app*.mp. 7186984

8 Humans/ or Wearable Electronic Devices/ or Monitoring, Ambulatory/ 19530772

9 1 or 2 or 3 or 4 or 5 or 6 or 7 or 8 24614336

10 monitor*.mp. 1042277

11 measur*.mp. 3866216

12 assess*.mp. 3592382

13 record*.mp 1255451

14 track*.mp 172040

15 captur*.mp. 209546

16 evaluat*.mp. 4174316

17 sens*.mp. 2505843

18 10 or 11 or 12 or 13 or 14 or 15 or 16 or 17 11366604

19 biomechanic$.mp. or Biomechanical Phenomena/ 159483

20 Gait Analysis/ or Gait/ 30543

21 kinematic$.mp. or Biomechanical Phenomena/ 138727

22 19 or 20 or 21 193785

23 home*.mp. 642967

24 community.mp. 614784

25 Remote Sensing Technology/ or remote.mp. 84956

26 free living.mp. 16497

27 portable.mp. 33174

28 23 or 24 or 25 or 26 or 27 1326588

29 9 and 18 and 22 and 28 4375

30 29 4375

31 limit 30 to (english language and yr="2015 -Current") 2084

**SCOPUS** Search Strategy results = 3885 Run 29.07.2021

TITLE-ABS-KEY ( tech* )  OR  TITLE-ABS-KEY ( device )  OR  TITLE-ABS-KEY ( mobile )  OR  TITLE-ABS-KEY ( internet )  OR  TITLE-ABS-KEY ( sens* )  OR  TITLE-ABS-KEY ( app* )  OR  TITLE-ABS-KEY ( wearable )  AND  TITLE-ABS-KEY ( measur* )  OR  TITLE-ABS-KEY ( assess* )  OR  TITLE-ABS-KEY ( track* )  OR  TITLE-ABS-KEY ( sens* )  OR  TITLE-ABS-KEY ( record* )  OR  TITLE-ABS-KEY ( evaluat* )  OR  TITLE-ABS-KEY ( monitor* )  OR  TITLE-ABS-KEY ( captur* )  AND  TITLE-ABS-KEY ( remote )  OR  TITLE-ABS-KEY ( home )  OR  TITLE-ABS-KEY ( communit* )  OR  TITLE-ABS-KEY ( free-living )  OR  TITLE-ABS-KEY ( portable )  AND  TITLE-ABS-KEY ( gait )  OR  TITLE-ABS-KEY ( biomechanic* )  OR  TITLE-ABS-KEY ( kinematic* )  AND  LANGUAGE ( english )  AND  PUBYEAR  >  2015

**PEDro** Search 23.08.2021

technolog* AND gait OR osteoarthritis AND technolog* from 2015 onwards = 41 results

# Excluded Criteria

**Table B - Exclusion Criteria**

| **Category** | **Reason** |
| --- | --- |
| Data / Metric | Outcome of the measures is not a specific kinematic or kinetic metric e.g. spatiotemporal, biomechanical, gait, force or pressure measure.  For example outcomes describing pattern of fall, quantity of physical activity, exercise adherence, self-reported / objective outcomes are excluded.  Modalities of the Activities of daily living ADL are measured e.g sitting, standing (not within a TUG) |
| Technology | Actual technology not specified e.g. paper relates to methodology only, application of an algorithm, or the hypothetical / theoretical capability of technology.  Technology was not validated against laboratory based existing ‘gold’ standard technology.  Technology already considered ‘gold standard’ as per list in table 1. |
| Population | Is not human or no human testing has taken place. |
| Context | Technology not suitable for use remotely: Remote is defined as being capable of being used outside of a traditional fixed laboratory setting.  Technology should be portable as a minimum and could be used in a clinical, community, home or ambulatory setting. |
| General | Is a systematic or scoping review.  Not a peer reviewed journal article. E.g. conference abstract  Published prior to 2015.  Not in English language. |
